# Comparing phthalate exposure between bottlenose dolphins (*Tursiops truncatus*) residing in urban and rural environments

**DOI:** 10.3389/fmars.2025.1554075

**Published:** 2025-05-11

**Authors:** Miranda K. Dziobak, Tita Curtin, Randall S. Wells, Ryan Takeshita, Cynthia R. Smith, Eric Zolman, Christina N. Toms, Robyn F. Allen, Leslie B. Hart

**Affiliations:** 1 Department of Health and Human Performance, School of Health Sciences, College of Charleston, Charleston, SC, United States; 2 Department of Environmental Health Sciences, Arnold School of Public Health, University of South Carolina, Columbia, SC, United States; 3 Brookfield Zoo Chicago’s Sarasota Dolphin Research Program, ℅ Mote Marine Laboratory, Sarasota, FL, United States; 4 Conservation Medicine, National Marine Mammal Foundation, San Diego, CA, United States; 5 Executive Operations, National Marine Mammal Foundation, San Diego, CA, United States; 6 Department of Psychology, New College Florida, Sarasota, FL, United States

**Keywords:** marine mammal, pollution, Sarasota Bay, Barataria Bay, plastic

## Abstract

**Introduction:**

Exposure to phthalate esters has previously been documented in bottlenose dolphins (*Tursiops truncatus*) inhabiting an urban estuary (Sarasota Bay, FL, USA; 2010–2019). Phthalates are chemicals commonly added to plastic products and consumer goods to enhance qualities such as flexibility, fragrance, and stability. Chemical leaching from products into the marine environment leaves wildlife vulnerable to reproductive, developmental, and metabolic impairment. Environmental phthalate exposure has been shown to vary relative to human activity and urbanization.

**Methods:**

To evaluate potential differences in dolphin exposure risk, urine was collected from free-ranging bottlenose dolphins residing in an urban (Sarasota Bay, FL, USA; 2010–2024; n=71) and rural estuary (Barataria Bay, LA, USA; 2011–2023; n=45). Urinary phthalate metabolite concentrations were quantified with high-performance liquid chromatography (HPLC; Agilent 1100; WatersXBridge BEH C18, 2.5 μm, 2.1×50 mm analytical column) coupled to a triple quadrupole mass spectrometer (MS; Applied Biosystems Sciex API 4000) with an electrospray ionization (ESI negative) interface.

**Results:**

The magnitude of MEHP detection did not differ significantly between sampling sites (p=0.97); however, MEHP was detected more frequently in Sarasota Bay dolphins (73.24%; n=52; 95% CI: 61.20–82.73) than Barataria Bay dolphins (33.33%; n=15; 95% CI: 20.00–48.95%). Dolphins from Sarasota Bay may be exposed to a greater diversity of phthalates compared to Barataria Bay dolphins, indicated by differences in the detected phthalate metabolite profile.

**Discussion:**

Notably, dolphins from Barataria Bay were impacted by the Deepwater Horizon oil spill, with evidence suggesting long-term negative health outcomes. The endocrine-disrupting effects of phthalates could exacerbate metabolic, reproductive, or immune dysfunction in dolphins, especially those with compromised health. The higher phthalate detection frequency in Sarasota Bay dolphins suggests increased urban exposure risks; however, detection in dolphins from Barataria Bay raises concerns for dolphins in recovering ecosystems. Further research is needed to assess potential synergistic impacts of chemical mixtures, and targeted mitigation strategies in contaminated environments.

## 1 Introduction

Phthalates are synthetic additives commonly used to increase plastic flexibility (Wormuth et al., 2006; ATSDR, 2019) and added to personal care products as solvents and stabilizers (ATSDR, 1995; Hauser and Calafat, 2005). There are approximately 21 commonly used phthalates that have a basic structure comprising two ester groups attached to consecutive carbons on a benzene ring (Zhang et al., 2015a). The most common phthalates observed in the marine environment are dimethyl phthalate (DMP), diethyl phthalate (DEP), di-n-butyl phthalate (DBP), benzyl butyl phthalate (BBP), and di(2-ethylhexyl) phthalate (DEHP; Gao and Wen, 2016). Phthalates are not chemically bound to the products to which they are added, allowing them to easily separate from the product and leach into the environment (Heudorf et al., 2007). For example, some phthalates (e.g., DEP, DEHP, and DBP) have been reported to leach out of materials composed of polyvinyl chloride (PVC) and polyethylene, as well as car parts, plastic toys, food packaging, paints, glues, and cosmetics (Paluselli et al., 2019; ATSDR, 2022).

Phthalates can enter the marine environment via degradation of plastic pollution (Paluselli et al., 2019), agricultural waste (Zhang et al., 2015a), commercial and industrial runoff and waste (Bergé et al., 2014; Langdon et al., 2019), or wastewater discharge (Clara et al., 2010). For example, Paluselli et al. (2019) observed the release of phthalates from fragments of polyethylene garbage bags, which continue to be a major contributor to marine plastic pollution (Suaria et al., 2020; Thushari and Senevirathna, 2020). In laboratory conditions, phthalates readily leach from plastic material into seawater, reaching peak concentrations within two weeks of introduction; light and bacteria increased aqueous concentrations 5-fold (Paluselli et al., 2019). Agricultural environments are often contaminated with phthalates, particularly during the summer months when plastic mulching films are used to protect crops and improve crop yield (Zhang et al., 2015b; Viljoen et al., 2023). Further, DEHP is a common component of most organic fertilizers (Zorníkováet al., 2014), and due to its low solubility in water, this phthalate can accumulate in agricultural soils (Cartwright et al., 2000; Niu et al., 2014). Additionally, environmental contamination from industrial and commercial sources can include leachate or runoff from improper disposal of electronic waste or parts (Liu et al., 2009). This is particularly true for phthalates that are more hydrophilic (e.g., MEP) and easily mobilize from the soil via leachate or runoff into nearby waterways (Langdon et al., 2019). Researchers have also examined environmental pollution relative to manufacturing plants. For example, high concentrations of DEHP have been found in the Houjing River at multiple sampling locations near two different industrial parks in southern Taiwan (20.22 mg/kg and 8.93 mg/kg, respectively; Kaewlaoyoong et al., 2018). Phthalates are also present in influent and effluent wastewater. Across 15 sampled wastewater treatment plants, DEP, DEHP, and BBP were present in 100% of influent wastewater samples, and DEHP and BBP remained present in 100% of treated wastewater samples while DEP remained present in 80% of treated samples (Clara et al., 2010).

Following exposure, mammals rapidly metabolize phthalates to their monoester form. Metabolism occurs through a series of steps, including phase I hydrolysis followed by phase II conjugation (Frederiksen et al., 2007; Choi et al., 2013). Long-branched phthalates, such as DEHP, often require further hydroxylation and oxidation before proceeding to the conjugation phase (Frederiksen et al., 2007). Generally, the more a compound is reduced, the less cytotoxic it becomes, which may be the impetus for metabolic rapidity (Staples et al., 1997). In the case of DEHP, however, metabolism increases toxicity (Gray and Gangolli, 1986; Fan et al., 2010; Gupta et al., 2010). Following metabolism, phthalate metabolites are excreted through urine and feces, where they are able to be detected and quantified (Frederiksen et al., 2007; Choi et al., 2013). Because of the metabolic process, phthalates are not expected to bioaccumulate (Staples et al., 1997; Heudorf et al., 2007); however, ongoing use and release of phthalates into the environment may result in chronic exposure opportunities for wildlife. In humans, urine is the preferred matrix for assessing phthalate exposure due to the reduced potential for contamination from the parent compound (Koch and Calafat, 2009) and the ability to detect metabolites in low concentrations (Blount et al., 2000).

Phthalate exposure has been implicated in many adverse health outcomes, including endocrine disruption (Monneret, 2017; Qian et al., 2020), reproductive impairment (Toft et al., 2012; Ye et al., 2014), and abnormal development (Lyche et al., 2009; Radke et al., 2019). The mechanism of endocrine disruption involves competitive inhibition, in which phthalates bind to endocrine receptors and prevent their target hormones from binding (Lee et al., 2013). The result is a disruption of the normal production, transport, and activity of hormones throughout the body. For example, BBP can bind to estrogen receptors and substantially reduce estrogenic activity (Lee et al., 2013), and DEHP can bind to thyroid signaling hormone receptors and result in hypothyroidism (Zhang et al., 2021). This endocrine disruption can lead to downstream reproductive impacts including infertility (Latini et al., 2003, 2006; Tranfo et al., 2012), poor semen motility (Jurewicz et al., 2013; Axelsson et al., 2015), decreased fecundity (Buck Louis et al., 2014; Seyoum and Pradhan, 2019; Yang et al., 2021), decreased testosterone levels (Kim et al., 2003; Meeker and Ferguson, 2014; Chang et al., 2015), and genital defects in males (Parks et al., 2000; Pant et al., 2014; Radke et al., 2018). For example, among men, exposure to DEHP, DBP, and DEP has been associated with decreased anogenital distance, reduced semen quality, and impacts on sperm motility (Pant et al., 2014; Radke et al., 2018; Yang et al., 2023). Among women, decreased fecundity (Hauser et al., 2016; Jukic et al., 2016), spontaneous abortion (Jukic et al., 2016; Messerlian et al., 2016), and preterm birth (Ferguson et al., 2014, 2019; Shoaff et al., 2016) were associated with exposure to certain phthalates, including DEHP, DBP, and DEP. Impacts on growth and metabolism are also reported, including an increased risk of obesity (Majeed et al., 2017; Stojanoska et al., 2017) and Type II diabetes (Chevalier and Fénichel, 2015; Zhang et al., 2023; Yang et al., 2025). These impacts are likely mediated through the thyroid as it is a target for phthalates, resulting in the abnormal production or secretion of hormones such as triiodothyronine (T_3_) thyroxine (T_4_), and insulin-like growth factor 1 (IGF-I;Boas et al., 2010).

Bottlenose dolphins (*Tursiops truncatus*) inhabiting Sarasota Bay, FL, USA have been the focus of several studies to understand phthalate exposure, given the embayment’s proximity to agricultural, industrial, and residential centers (Hart et al., 2018; Dziobak et al., 2021, 2022a). Nearly 75% of sampled dolphins (*n* = 51) were exposed to at least one type of phthalate, and exposure was not limited to a particular sex or age class (Dziobak et al., 2021). Among the eight compounds screened (monomethyl phthalate (MMP), monoethyl phthalate (MEP), mono (2-ethylhexyl) phthalate (MEHP), mono(2-ethyl-5-oxohexyl) phthalate (MEOHP), mono(2-ethyl-5-hydroxyhexyl) phthalate (MEHHP), monobenzyl phthalate (MBzP), monobutyl phthalate (MBP), and mono-isobutyl phthalate (MiBP)), the most commonly detected metabolites were MEP and MEHP (Hart et al., 2018; Dziobak et al., 2021), which are metabolites of parent compounds commonly added to personal care products (Hauser and Calafat, 2005) and plastic goods, respectively (Paluselli et al., 2019). When compared to data collected as part of the Centers for Disease Control and Prevention’s (CDC) National Health and Nutrition Examination Survey (NHANES), Sarasota bottlenose dolphin urinary concentrations of MEHP were significantly higher than human samples (Hart et al., 2020). While the exact sources and implications of this exposure are currently unknown for Sarasota Bay dolphins, there is evidence of plastic ingestion (particularly transparent films and white foams; Hart et al., 2022) and thyroid disruption (Dziobak et al., 2022b).

Barataria Bay, LA, USA, is located in the northern Gulf of Mexico, approximately 80 miles south of New Orleans, LA, USA. The bay is bordered by mainland Louisiana to the north and Grand Isle to the south. Compared to the urban environment and coastal development of Sarasota Bay, Barataria is a more remote location with a much smaller human population primarily restricted to the barrier island of Grand Isle. In 2010, an offshore oil rig exploded in the Gulf of Mexico causing the largest domestic marine oil spill in history (*Deepwater Horizon*; Liu et al., 2011), and bottlenose dolphins in Barataria Bay have since been the focus of long-term monitoring to assess the impacts of oil exposure on health (Schwacke et al., 2017; Smith et al., 2017), movement (Takeshita et al., 2021), and survival (McDonald et al., 2017; Schwacke et al., 2022). This long-term monitoring has included catch-and-release health assessments, providing the opportunity to collect urine samples from free-ranging dolphins.

Phthalate use is extensive, and contamination has been documented in every environmental matrix (e.g., air; Adibi et al., 2008; Lee et al., 2019; sediment; Arfaeinia et al., 2019; Lee et al., 2019; Weizhen et al., 2020; biota; Valton et al., 2014; Fourgous et al., 2016; Baini et al., 2017; Hart et al., 2018; freshwater; (Fatoki et al., 2010; Cheng et al., 2019); seawater; Zhang et al., 2018) across urban and rural locations (Wang et al., 2014); however, the distribution of phthalates has been shown to vary across locations. For example, studies conducted in China have observed increased phthalate pollution along a rural-urban gradient, with the highest concentrations found in the most urbanized areas (Lan et al., 2012; Hongjun et al., 2013). These findings are supported by other studies that detected increased phthalate concentrations in urban and industrialized areas compared to rural (Sun et al., 2013; Wang et al., 2018; Karamianpour et al., 2023). Notably, many studies of phthalates in soils or sediments that documented differences between sites still had detection in the majority of all samples, highlighting the ubiquity of environmental phthalate pollution (Sun et al., 2013; Duong et al., 2014). Similar relationships between phthalate exposure and urbanization have been detected in humans, where urban human populations have higher phthalate metabolite concentrations compared to rural locations (Prasad et al., 2022; Runkel et al., 2022). This is also mirrored in aquatic environments, potentially due to phthalate contamination discharged from industrial effluents and untreated wastewater (Zhang et al., 2015b).

Given widespread phthalate pollution in the environment, contamination is expected for dolphins residing in both urban and rural locations, though the variation between sites is currently unknown. Therefore, the objective of this study is to characterize and compare phthalate exposure among urban Sarasota Bay, Florida dolphins compared to rural Barataria Bay, Louisiana dolphins to better understand the impacts of human activity on environmental pollution and subsequent exposure.

## 2 Methods

### 2.1 Site selection

Dolphins sampled for this study were from the Barataria Bay Estuarine System Stock occupying Barataria Basin as well as dolphins from the National Marine Fisheries Service (NMFS) stock that includes dolphins using SSB, and Little SSB (Tyson and Wells, 2016). Although both locations are estuarine systems, the barrier islands of BAR are much less populated than the city of Sarasota (1,005 residents compared to ~55,000, respectively (US Census Bureau, 2024b, a). Further, land bounding the Barataria Basin is primarily privately owned, with 22% of the basin developed (CWPPRA, 2022), while land in Sarasota County is zoned for agricultural, residential, commercial, and industrial use (City of Sarasota, 2024). Therefore, dolphins residing in BAR were identified as “rural” while dolphins residing in SSB were identified as “urban”. However, it should be noted that BAR has a high concentration of petrochemical infrastructure and activity, with most operations based out of Grand Isle and Port Fourchon. Dolphins from both BAR and SSB are known to exhibit year-round site fidelity (Wells, 2009; McDonald et al., 2017), thus contaminant exposure is expected to reflect their local environments.

### 2.2 Urine collection

**FIGURE 1:**
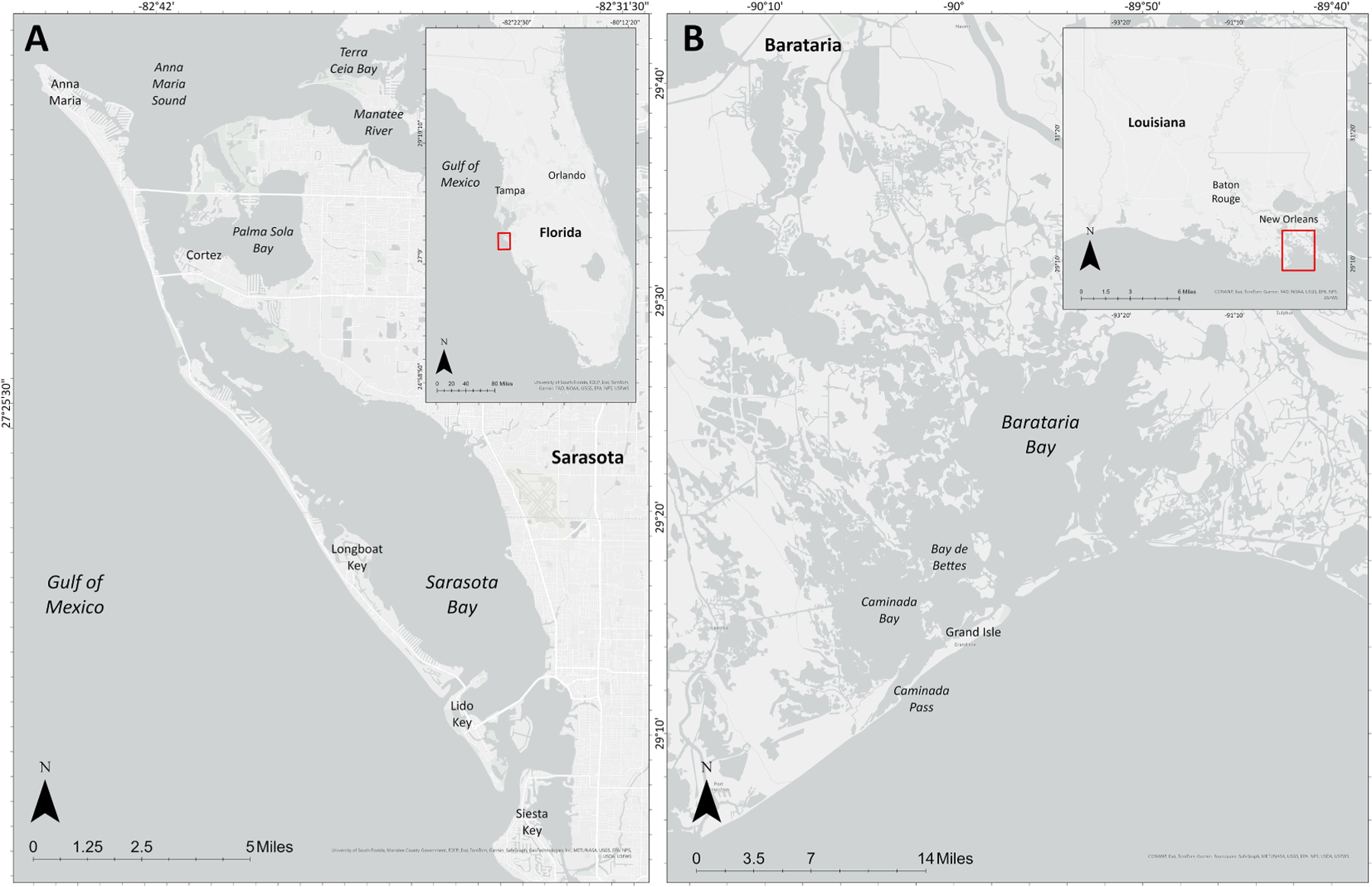
Bottlenose dolphins study sites: **(A)** Sarasota Bay, Florida and **(B)** Barataria Bay, Louisiana. Map created using Esri ArcGIS Pro basemap, Esri, TomTom, Garmin, FAO, NOAA, USGS, ^©^ OpenStreetMap contributors, and the GIS User Community.

Dolphins sampled for this study were individuals considered to be resident to Sarasota Bay, Florida (SB; n~170; Wells, 2009; Lacy et al., 2021) and Barataria Bay, Louisiana (BB; n~2,000; Mullin et al., 2018; Figure 1). Urine was collected opportunistically via catheterization from dolphins sampled during catch-and-release health assessments. For BAR dolphins, urine was collected via urinary catheterization, using an 8.5Fr 60 cm multipurpose drainage catheter (Cook Medical, Bloomington, Indiana) for males and a 10.2Fr 45 cm multipurpose drainage catheter (CookMedical) or a 10Fr 90 cm Foley catheter (Mila International, Erlanger, Kentucky) for females. For SSB dolphins, urine was collected either from free catch using a 50 ml conical vial or via urinary catheterization, using a lubricated (surgilube) 8Fr 22 in (adults) or 5Fr 16 in (juveniles) Kendall Sovereign Feeding Tube and Urethral Catheter (Patterson Veterinary, Loveland, CO). For both locations, samples were frozen with liquid nitrogen for transport in vapor shippers and stored below −20°C until analysis. Health assessments were conducted in BAR during 2011, 2013–2014, 2017–2018, and 2023 as a result of the *Deepwater Horizon* oil spill, as well as routinely in SSB during 2010–2019, 2022–2024. Some dolphins (n=15) were sampled repeatedly during these health assessments; however, repeated samples were not included in analysis. Health assessment methods have been described elsewhere (Wells et al., 2004, Barratclough et al., 2019). Briefly, dolphins were encircled and temporarily restrained to collect a variety of biological, physiological, and morphological samples/data. Health assessments in BAR were conducted under NMFS Scientific Research Permits Nos. 932–1905, 18786, and 24539. Health assessments in SSB were conducted under NMFS Scientific Research Permits Nos. 522–1785, 15543, 20455, and 26622.

### 2.3 Sample processing and analysis

Each urine sample was screened for seven phthalate metabolites (MEHP, MEHHP, MEOHP, MBzP, MiBP, and MBP). Male samples containing sperm were centrifuged at 3,000 rpm for 5 minutes prior to solid phase extraction (SPE) to separate the urine from the sperm and prevent clogs in the SPE cartridge. Urine samples (1 mL) were spiked with isotopically labeled internal standards and extracted via SPE (Agilent Bond Elute Nexus) and quantified with high-performance liquid chromatography (HPLC; Agilent 1100; WatersXBridge BEH C18, 2.5 μm, 2.1 × 50 mm analytical column) coupled to a triple quadrupole mass spectrometer (MS; Applied Biosystems Sciex API 4000) with an electrospray ionization (ESI negative) interface. Sample integrations were performed using Analyst software (Sciex, ver 1.5). Prior to the acquisition of sample data, the instrument was calibrated (standard reference material (SRM) 3060: monoester phthalates in acetonitrile); coefficients of determination (r2) for all metabolites were ≥ 0.995.

As reported by Hart et al. (2018), quality assurance/quality control (QA/QC) samples (reagent blanks, reagent spikes, matrix spikes, SRM 3672 Organic Contaminants in Smokers’ Urine, and field blanks) were processed alongside the urine samples. Reagent blank values were subtracted from the determined concentration value to account for any metabolite contamination resulting from laboratory processes. Available field blank metabolite concentrations were not found to be statistically different from each other by year or by metabolite. Field blank concentrations were averaged for each metabolite and subtracted from urine samples for any contamination due to sample collection materials (e.g., catheters). Acceptable QA/QC criteria for spike (reagent and matrix) and SRM recoveries were 70%−130%. The limit of detection (LOD) was determined for each metabolite and is based on the lowest point on the calibration curve that could be detected on the instrument divided by the volume of the sample extracted (Dziobak et al., 2025).

### 2.4 Statistical analysis

Descriptive statistics were used to summarize phthalate metabolite concentrations and calculate the proportion of dolphins with concentrations above the LOD (“detectable concentrations”; Dziobak et al., 2025. For dolphins sampled more than once, only the sample most recently obtained was used for analysis. All statistical analyses were conducted using R (Version 4.3.2, R Foundation for Statistical Computing, Vienna, Austria) and R Studio (Posit Software, BBC) software packages. For metabolites with greater than 20% of concentrations above LOD, means and standard deviations were calculated using robust regression on order statistics (Helsel, 2006) using the NADA (Lee and Helsel, 2022) and NADA2 (Julian and Helsel, 2023) packages. Previous work demonstrated no demographic differences in phthalate metabolite detection, but used a small sample size (Dziobak et al., 2021). Therefore, summary statistics were calculated overall and by demography (female, male, immature, and mature) within each site to retest relationships with a larger sample size. Immature dolphins were differentiated from mature dolphins on the basis of sexual maturity, which was determined based on several measures, including age, calving history, pregnancy diagnosis via ultrasonography, testis size from ultrasound, and sex hormone concentrations (Wells et al., 1987, 2025; Wells, 2014). Dolphin ages were determined by either life history from photo-identification (id) surveys, analysis of dentinal growth layer groups (Hohn et al., 1989), or analysis of pectoral flipper radiography (Barratclough et al., 2019). The proportion of detectable concentrations was compared between demographic groups and between sites using a Peto-Peto test (NADA and NADA2 R packages; (Helsel, 2006; Dziobak et al., 2021). Among dolphins with detectable concentrations, distribution of concentrations was evaluated using a Shapiro-Wilk test, and means were compared between sites using either an independent t-test or a Mann-Whitney U test, depending on Gaussian distribution. Statistical significance in observed differences was determined using α = 0.05.

### 2.5 Spatial analysis

Sighting data from BAR were not abundant (compared to SSB, for example) as routine photo-id surveys are not conducted. Instead, location data resulted from a variety of efforts, including photo-id surveys, biopsy locations, radio tracking, stranding locations, health assessment locations, satellite-linked telemetry, and fecundity studies. Because some BAR dolphins had very few observations (some fewer than 10), all available records were used to estimate spatial usage. Sighting data for dolphins residing in SSB was obtained from standardized photo-id surveys (Wells, 2009; Tyson and Wells, 2016) conducted by the Sarasota Dolphin Research Program (SDRP) and included more than 20,000 individual sighting records. In humans, phthalate metabolism is rapid; daily fluctuations have been observed in urine samples (Hauser et al., 2004; Teitelbaum et al., 2008). However, single spot samples can be used as a predictive metric to obtain average metabolite concentrations representative of the previous 3–6 (Hauser et al., 2004; Teitelbaum et al., 2008). Therefore, SSB sighting data were restricted to one calendar year prior to urine sampling to generate the most conservative spatial use estimate for the dolphins.

Spatial analyses were performed to compare spatial usage between dolphins with detectable urinary metabolite concentrations (hereafter referred to as “detects”) and dolphins with urinary metabolite concentrations below LOD (hereafter referred to as “nondetects”) to identify potential areas of phthalate contamination in Barataria and Sarasota Bays. Spatial analyses were based on methods described in Takeshita et al. (2021). Briefly, kernel density estimates (KDEs) were calculated using the *adehabitatHR* package in R (Calenge, 2006), to determine 50% and 95% percent volume contours (PVCs) which represent the core (50% PVC), and total (95% PVC) area usage for detects and nondetects. The bandwidth parameter was determined using a rule-based *ad-hoc* approach (Kie, 2013).

## 3 Results

### 3.1 Barataria Bay

**FIGURE 2:**
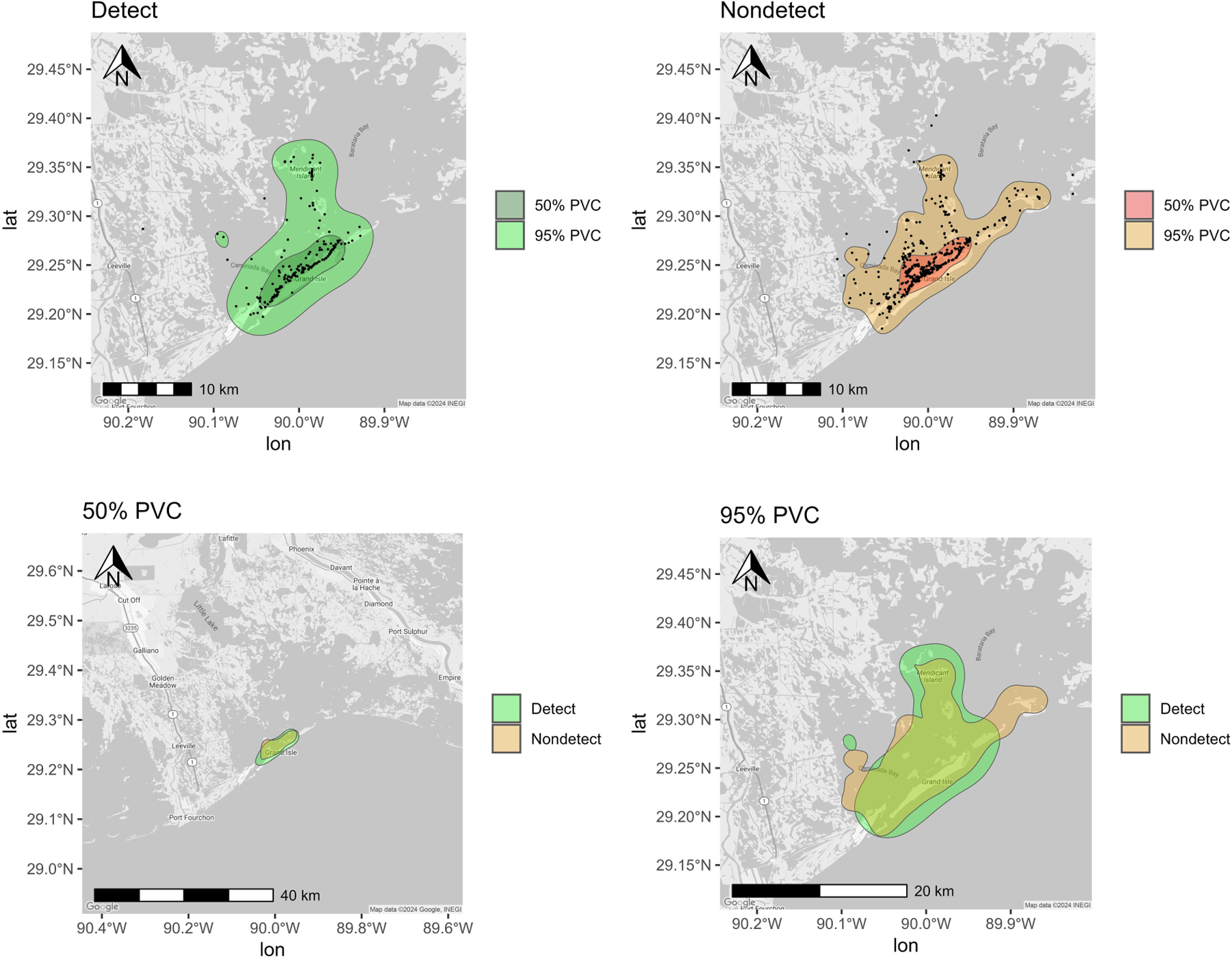
Percent volume contours (PVCs) determined for bottlenose dolphins sampled (n = 45) from Barataria Bay (2011, 2013–2014, 2017–2018, 2023) using all sighting histories. (Detect and Nondetect maps) Core (50% PVC) and total (95% PVC) spatial usage respectively for dolphins with detectable and undetectable urinary mono(2-ethylhexyl) phthalate MEHP concentrations with black points representing individual sighting records. (50% PVC and 95% PVC maps) Core (50% PVC) and Total (95% PVC) spatial usage respectively for dolphins with detectable urinary MEHP concentrations overlayed with spatial usage for dolphins with nondetectable urinary MEHP concentrations.

Urine for phthalate metabolite analysis in BAR was collected from 45 individual dolphins sampled during catch-and-release health assessments conducted in Barataria Bay, LA, USA in 2011, 2013, 2014, 2017, 2018, and 2023 (Table 1; Figure 2). Detectable concentrations of at least one metabolite were observed in 33.33% (n = 15; 95% CI: 20.00 – 48.95%) of samples. Metabolite detection was limited to three compounds including MEHP, MBzP, and MEHHP; none of the dolphins sampled had detectable concentrations of MBP, MEOHP, MEP, or MiBP (Table 1). The most commonly detected metabolite was MEHP (n = 14) with concentrations ranging from 1.40 µg/L to 16.46 µg/L (Tables 1, 2). Given low detection frequencies for the majority of the metabolites, MEHP was the only metabolite that was statistically evaluated for differences in detection relative to demographic characteristics.

There was a nearly even distribution of sexes (23 female, 22 male) and age classes (21 immature, 24 mature) sampled (Table 2). The majority of samples were collected in 2011 (n =14), and subsequent years exhibited variability in the number of samples screened (ranging from 2 to 8 samples; Table 2). Regardless, phthalate metabolites were detected in at least one individual from each sampling year except 2013 (Table 2). There were no significant differences in the percentage of females (26.09%; 95% CI: 10.23–48.41%) and males (36.36%; 95% CI: 17.20–59.34%) with detectable concentrations of MEHP (p=0.50; Table 2). Mean concentrations of MEHP for females and males were 1.10 µg/L (s.d. = 1.70 µg/L) and 2.62 µg/L (s.d. = 4.48 µg/L; Table 2), respectively. Similarly, there were no significant differences in the percentage of sampled dolphins with detectable MEHP between mature and immature dolphins (33.33% vs. 29.17%; p=0.80; Table 2). Both age classes had the same mean concentration (immature dolphins: 1.81 µg/L, s.d. = 3.14 µg/L; mature dolphins: 1.81 µg/L, s.d. = 3.80 µg/L; Table 2).

MEHP was the most frequently detected metabolite in BAR dolphin urine samples, so variations in bay usage were compared between dolphins with MEHP concentrations above LOD (“detects”) and equal to or below LOD (“nondetects”). Overall little variation in habitat usage was observed in BAR between detects and nondetects (Figure 2). Both had core (50%) ranging patterns around the barrier islands (e.g., Grand Isle), and total (95%) ranging patterns that extended into BAR (e.g., Caminada Bay; Figure 2). Nondetects appeared to have a range that extended further east past Grand Isle towards Grand Terre than detects, who were not sighted past Barataria Pass (Figure 2).

### 3.2 Sarasota Bay

**FIGURE 3:**
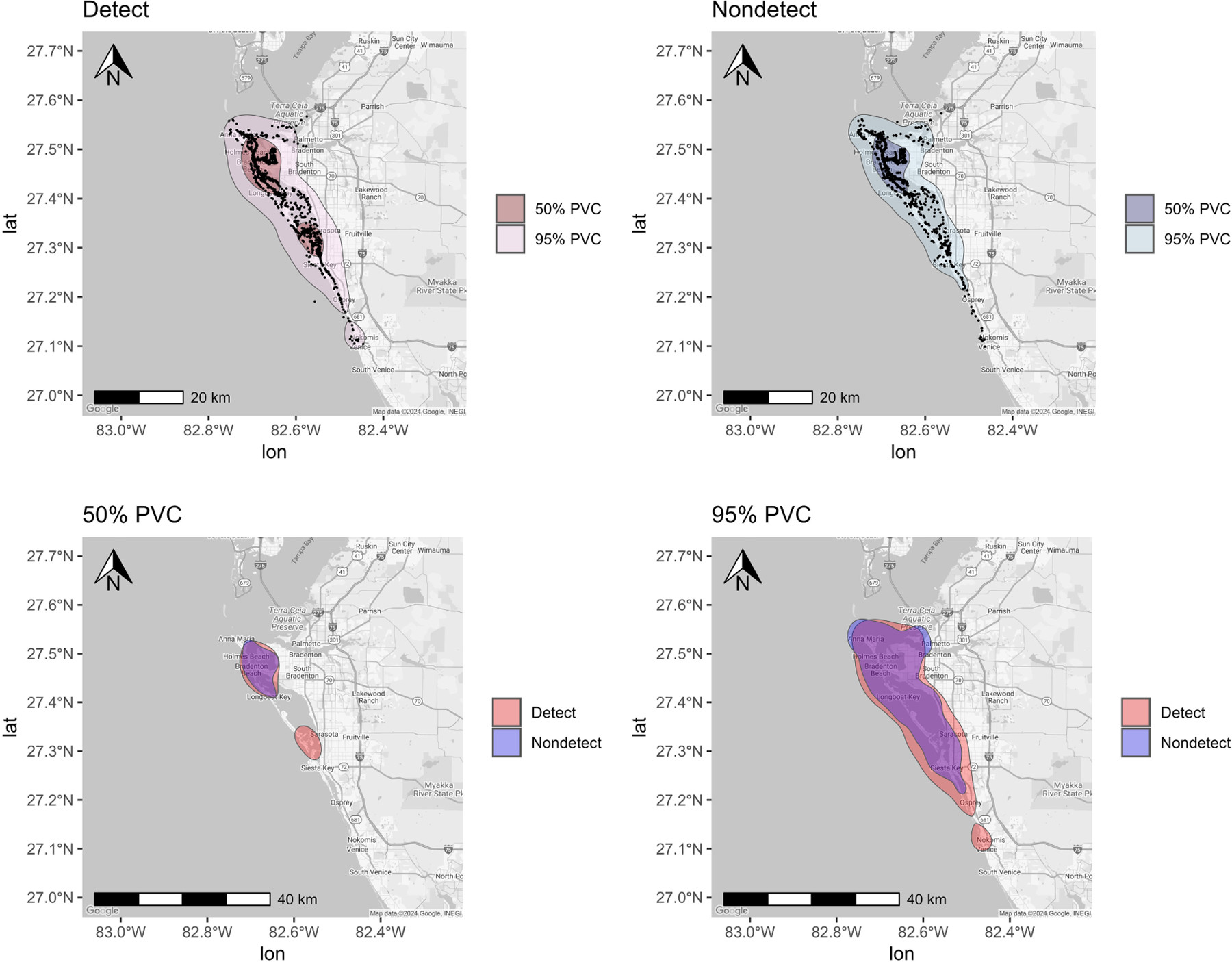
Percent volume contours (PVCs) determined for bottlenose dolphins sampled (n = 71) from Sarasota Bay (2010–2019, 2022–2024) using photo-identification sighting histories. (Detect and Nondetect maps) Core (50% PVC) and total (95% PVC) spatial usage respectively for dolphins with detectable and undetectable urinary mono(2-ethylhexyl) phthalate MEHP concentrations with black points representing individual sighting records. (50% PVC and 95% PVC maps) Core (50% PVC) and Total (95% PVC) spatial usage respectively for dolphins with detectable urinary MEHP concentrations overlayed with spatial usage for dolphins with nondetectable urinary MEHP concentrations.

Urine for phthalate metabolite analysis in SSB was sampled from 71 individual dolphins during catch-and-release health assessments conducted during 2010–2019, and 2022–2024 (Figure 3), of which 73.24% (n= 52; 95% CI: 61.41–83.06%); had concentrations of at least one metabolite above LOD. Among dolphins with detectable concentrations, one to five metabolites were detected. Six of the seven metabolites screened for were detected, including MBP, MBzP, MEHHP, MEHP, MEOHP, MEP (Table 3). Similarly to previous studies of phthalate exposure in SSB dolphins, the most common metabolites detected were MEHP (n=41, 57.75%; 95%CI: 45.44–69.39%) and MEP (n=13, 18.31%; 95% CI: 10.13–29.27%), and none of the dolphins had detectable concentrations of MiBP (Table 3). Similar to samples from BAR, MEHP was the only metabolite analyzed for demographic differences as the other metabolites had too low detection frequencies.

The demographic distribution of dolphins was similar by sex (38 female, 33 male); however, there were almost twice as many mature dolphins (n = 44) than immature dolphins (n = 27). Across years, there were variations in the number of dolphins sampled, ranging from 1 (2013) to 15 (2024). There were no significant differences in the proportion of dolphins with detectable MEHP concentrations by sex (63.16% of females; 95% CI: 45.99 – 78.19%; 51.52% of males; 95% CI: 33.54–69.20%; Table 4) or by age class (62.96% of immature dolphins; 95% CI: 42.37–80.60%; 54.55% of mature dolphins; 38.85–69.61%; Table 4). The mean MEHP concentration for females was 9.77 µg/L (s.d. = 16.20 µg/L; Table 4), which was higher than males (3.74 µg/L; s.d., = 9.90 µg/L; Table 4). The mean MEHP concentration was also higher in mature dolphins (9.36 µg/L; s.d. = 16.60 µg/L; Table 4) compared to immature dolphins (3.11 µg/L; s.d. 6.20 µg/L; Table 4).

Since MEHP was also the most frequently detected metabolite in SSB, variations in bay usage were evaluated between detects and nondetects. Detects appeared to be more likely to use more of SSB than nondetects, as illustrated by a larger total area (95% PVC) used (Figure 3). Areas associated with detects included Anna Maria Sound, Palma Sola Bay, and part of SSB, as well as between New Pass and Venice Inlet (Figure 3). Although nondetect ranges were similar to detects in the northern part of the bay, the southern extension seemed to be limited to Siesta Key and did not go as far as Venice Inlet (Figure 3). Further, detects had core (50%) ranging patterns that were observed in the southern part of the bay near Venice, while nondetects were concentrated around Palma Sola Bay (Figure 3). Overall, the dolphin ranging patterns seemed to be consistent with ranging patterns previously reported (Dziobak et al., 2022a), where detects were more likely to be found in the southern portion of the bay compared to nondetects. Both groups, however, were likely to use the northern portion of the bay near Palma Sola (Figure 3), which was consistent with findings from a previous study (Dziobak et al., 2022a).

### 3.3 Barataria Bay vs. Sarasota Bay

**FIGURE 4:**
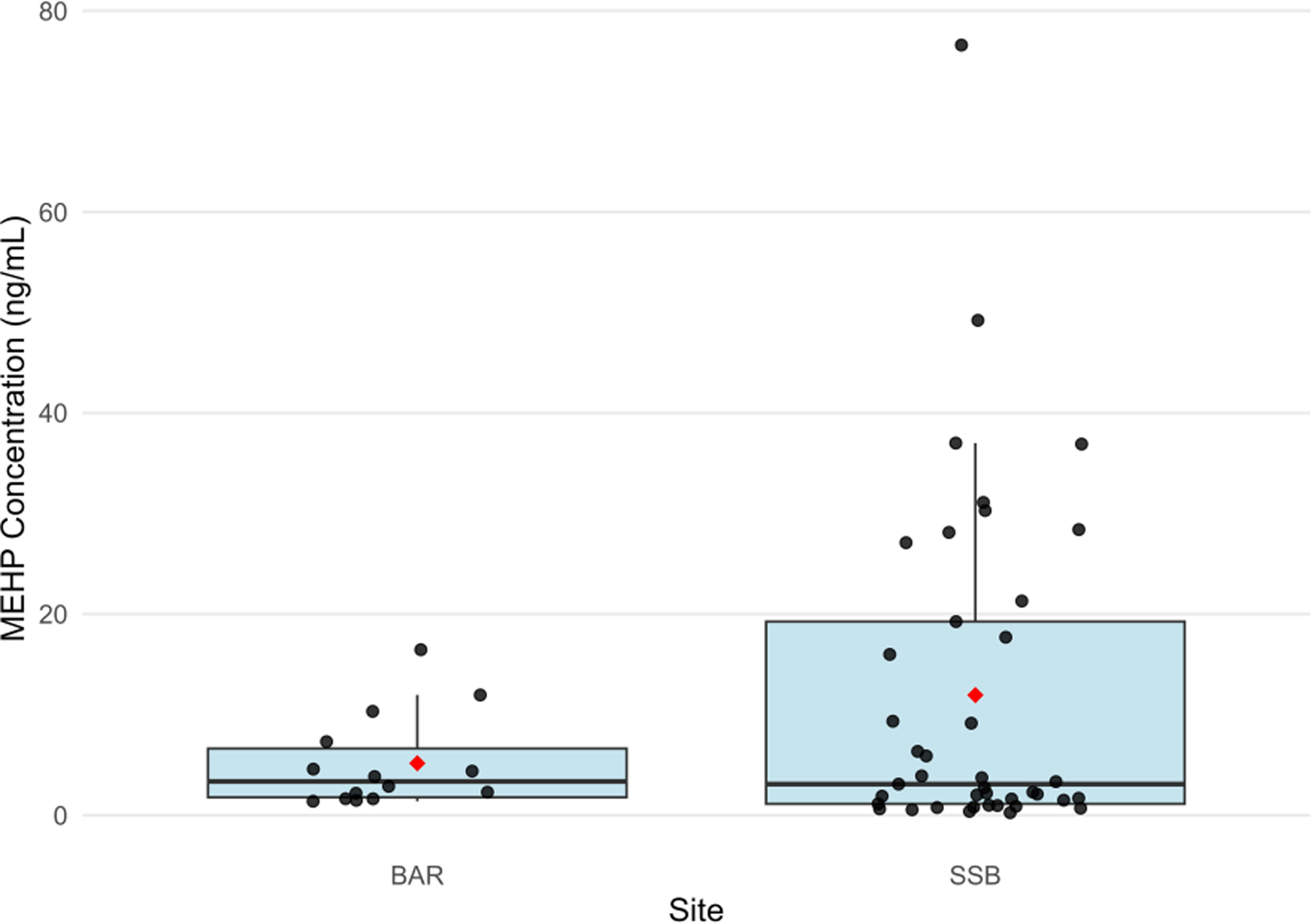
Comparison of urinary MEHP concentrations (>LOD) between bottlenose dolphins sampled in Barataria Bay, LA (BAR) and Sarasota Bay, FL (SSB). Box plot displays median and quartiles, black points represent concentrations above the limit of detection, and red diamond represents mean among concentrations above the limit of detection.

To explore the potential influence of urbanization on phthalate exposure, urinary MEHP concentrations were compared between BAR and SSB dolphins. MEHP was detected more frequently amongst SSB dolphins (n=41; 57.75%; 95% CI: 45.44–69.39%) than BAR dolphins (n = 14; 31.11%; 95% CI: 18.17–46.65%; p=0.005; Table 5). However, detectable concentrations of urinary MEHP did not differ significantly by site (BAR MEHP mean (s.d.) = 5.18 (4.66) µg/L; SSB MEHP mean (s.d.) = 11.95 (16.67) µg/L; p=0.97; Table 5, Figure 4), indicating similarities in the magnitude of DEHP exposure.

## 4 Discussion

### 4.1 Phthalate exposure

Of the 45 individual BAR dolphins screened, one-third had detectable concentrations of at least one phthalate metabolite in their urine. Our findings demonstrate a lower level of phthalate exposure in BAR dolphins compared to a previous study in bottlenose dolphins living near an urban environment with extensive coastal development (Dziobak et al., 2022a). Although there is fishery and petrochemical activity throughout the Barataria estuarine system, the smaller coastal community of residents, workers, and tourists is mostly concentrated around Grand Isle. Most of the dolphins in this study were assessed near Grand Isle and likely spend much of their time near the barrier islands, however some of the animals likely show high site fidelity to the northern, less developed areas of Barataria Basin (Figure 2; Takeshita et al., 2021).

Among bottlenose dolphins sampled in SSB, phthalate metabolites were detected in 73% of urine samples (95% CI: 61.20–82.73). SSB dolphins exhibited exposure to a diverse array of phthalates, with detectable concentrations determined for six of the seven metabolites screened. These metabolites come from parent compounds commonly added to plastic products (e.g., DEHP, DBzP) and personal care products (e.g., DEP). Consistent with previous investigations of SSB dolphins, MEHP was the most frequently detected phthalate metabolite. The majority of land around the SSB watershed is considered to be built up (USF, 2020). Urbanization has been linked with increased environmental phthalate pollution (Teil et al., 2014), so the development and human activity in the SSB area could contribute to marine contamination.

### 4.2 Comparison of phthalate exposure between Sarasota Bay and Barataria Bay dolphins

Although differing in the frequencies of detection, dolphins from both SSB and BAR exhibited exposure to phthalates, as evidenced by the detection of MEHP, MEHHP, and MBzP. The parent compounds (i.e., DEHP and BzBP) are most commonly added to plastic, which could be the exposure source. When studied in seawater conditions, plastics degraded by at least 85%, leaching large concentrations of these compounds into the aquatic environment (Paluselli et al., 2019). For example, MEHP was detected the most frequently in BAR dolphins in 2011, which coincided with cleanup efforts from the *Deepwater Horizon* oil spill. Materials deployed during this time period included polypropylene-filled sorbent booms (Kokaly et al., 2013; Blum et al., 2014) and containment booms, which can be composed of polypropylene, polyurethane, nylon, and PE (Schrader, 1991; Dadhich and Lalu, 2018; Pagnucco and Phillips, 2018). Phthalates such as DEHP, DMP, DEP, DiBP, and DnBP are known to leach from these synthetic materials (Paluselli et al., 2019; Zhong et al., 2023) and could have contributed to the increased exposure observed in BAR dolphins.

Beyond the materials used in BAR, plastic contamination in the ocean is extensive, with global abundance estimated at 82–358 trillion plastic particles weighing as much as 4.9 million tonnes (Eriksen et al., 2023). Regardless of worldwide ubiquity, contamination skews higher in urban regions with extensive coastal development (Kwon et al., 2020; Wardlaw et al., 2022). For example, Wardlaw et al. (2022) reported a higher concentration of microplastic fragments and fibers in urban waters compared to rural waters of the Thames River in Ontario, Canada (31–1882 fragments/kg sediment, 15–562 fibers/kg sediment in urban areas, 7–46 fragments/kg sediment, 46–216 fibers/kg sediment in rural areas). Similarly, urban waters of coastal South Korea exhibited higher concentrations of microplastic pollution (2.85 particles/m^3^) than rural waters (1.86 particles/m^3^; Kwon et al., 2020). MEHP was detected in BAR dolphins at similar concentrations to dolphins in SSB (Table 5), but the proportion of detects was lower (Table 5, Figure 4), possibly due to differences in land use and development between the two locations. As BAR is relatively remote and has little commercial or residential development use, there are likely fewer sources of plastic pollution compared to SSB. Further, BAR is much larger, covering over 1,600 km^2^ compared to 114.00 km^2^ covered by SSB (GulfBase, 2024a, b). Therefore, it is possible that any phthalate inputs to BAR are diluted throughout the bay prior to dolphin exposure.

Phthalate contamination may be more diverse in urban areas compared to areas with less coastal development. MEHP, MBzP, and MEHHP were detected in dolphins from both locations, while MEP and MBP were exclusively detected in SSB dolphins. In particular, MEP was the second most common phthalate metabolite detected in SSB dolphin urine samples (% detect). The parent compound, DEP, is commonly used as a fragrance enhancer for a variety of consumer products, including personal care and cleaning products, laundry detergents, and pet cleansers (Hubinger, 2010; Guo and Kannan, 2013; Guo et al., 2014). As a result, DEP is often found in industrial and domestic effluents (Bergéet al., 2014; Sengupta et al., 2014). Several studies have demonstrated a correlation between levels of DEP in wastewater and proximity to urban development (González-Mariño et al., 2017; Du et al., 2018; Li et al., 2020). Li et al. (2020) observed a strong relationship between DEP and industrial development, where lakes adjacent to industrial activity demonstrated higher median concentrations (14.728 µg/L) than lakes in areas with little industrial development or land use (8.684 µg/L). Similarly, González-Mariño et al. (2017) observed that DEP was the most abundant phthalate in residential wastewater, with a significantly higher average daily exposure load compared to all other measured phthalates. This was further supported by Du et al. (2018), who observed a positive correlation between population density and levels of DEP in samples collected from wastewater treatment plants. Remote areas often lack the commercial and industrial infrastructure that introduces phthalates into the environment. Given the remote location of BAR and the surrounding small population, the lack of MEP detection among dolphins in this rural area may further suggest a relationship between environmental DEP contamination and human activity.

### 4.3 Significance

The number of dolphins with evidence of overall phthalate and MEHP exposure in BAR was not as great as it was in SSB; however, the magnitude of exposure in phthalate-positive dolphins was consistent across sites (Table 5, Figure 4). Hart et al. (2020) found significantly higher urinary MEHP concentrations in SSB dolphins compared to human reference populations, so these findings suggest that BAR dolphin exposure may also be higher than values reported by the Centers for Disease Control and Prevention. MEHP has been associated with reproductive, metabolic, and developmental health effects in humans (Boas et al., 2010; Toft et al., 2012; Tranfo et al., 2012; Pant et al., 2014; Radke et al., 2019), marine mammals (Routti et al., 2021; Dziobak et al., 2022b), fish (Mathieu-Denoncourt et al., 2015), and laboratory rodent studies (Weaver et al., 2020; Wang et al., 2021; Adam and Mhaouty-Kodja, 2022). Given that the health of BAR dolphins is already compromised due to oil and associated chemical exposure, exposure to MEHP could exacerbate pre-existing conditions such as impaired stress response, suppressed endocrine activity, inflammation, and hypoglycemia (Schwacke et al., 2014; Smith et al., 2017) and further impact reproductive success (Kellar et al., 2017) and population recovery (Schwacke et al., 2024).

The types of phthalate metabolites observed in biological and environmental samples may reveal sources of contamination or environmental stressors. For example, Li et al. (2020) screened for phthalates in lakewater collected from 16 Chinese provinces with varying degrees of industrial and agricultural activity. While there was considerable overlap in the mixture of metabolites detected in water regardless of land use, DIBP concentrations were significantly higher in lakes adjacent to industrial sites, suggesting that detection of this particular phthalate could be indicative of certain human activities (Li et al., 2020). Findings from our study suggest that MEP could similarly characterize coastal or marine ecosystems that differ in the level or types of anthropogenic influence. MEHP has been observed in marine studies globally, including the United States (Brock et al., 2016; Hart et al., 2018; Dziobak et al., 2021), France (Valton et al., 2014; Fourgous et al., 2016), China (Hu et al., 2016), Italy (Fossi et al., 2012), and the Mediterranean Sea (Baini et al., 2017). Given that DEHP is predominately added to plastic products and that our oceans are estimated to contain more than 170 trillion plastic particles (Eriksen et al., 2023), we hypothesize that widespread detection of MEHP reflects the global ubiquity of plastic pollution.

## 5 Conclusion

Exposure to phthalates was compared between dolphins residing in different geographic regions. Although the magnitude of exposures were similar, dolphins in urban SSB had a higher frequency of phthalate metabolite detection compared to rural BAR (p = 0.005). Specific exposure sources are still widely unknown, but the metabolites detected could provide clues as to origin. For example, MEHP was found in both locations, suggesting evidence of widespread plastic pollution, while MEP detected only in Sarasota Bay dolphins could indicate relationships with human activity and urbanization. Widespread phthalate contamination and subsequent exposure is concerning due to endocrine disrupting properties, as well as the potential for cardiovascular and neurological impacts. Further research is needed to better understand phthalate exposure sources and associations with markers of dolphin health.

## Figures and Tables

**TABLE 1:** Frequency of detection of all metabolites in urine samples collected from bottlenose dolphins (*Tursiops truncatus*) in Barataria Bay, Louisiana (n=45; 2011, 2013, 2014, 2017, 2018, 2023).

	MBP	MBzP	MEHHP	MEHP	MEOHP	MEP	MiBP
# Above LOD	0	1	1	14	0	0	0
% Above LOD (95% CI)	0.00	2.22 (0.06–11.77)	2.22 (0.06–11.77)	31.11 (18.17–46.65)	0.00	0.00	0.00

**TABLE 2:** Frequency of detection, ROS mean and standard deviation, minimum, and maximum values of mono-2-ethylhexyl phthalate (MEHP) above the limit of detection (LOD; “detects”) by demography in bottlenose dolphins sampled in Barataria Bay during 2011, 2013, 2014, 2017, 2018, 2023.

Variable	Total (n=45) n (% of Total)	MEHP detects (n=14) n (%; 95% CI)	p^1^	Mean MEHP (s.d.)^2^ (µg/L)	Minimum MEHP (µg/L)^3^	Maximum MEHP (µg/L)^3^
Sex			0.50			
Female	23 (51.11)	6 (26.09; 10.23–48.41)		1.10 (1.70)	1.64	7.32
Male	22 (48.89)	8 (36.36; 17.20–59.34)		2.62 (4.48)	1.40	16.46
Age Class			0.80			
Immature	21 (46.67)	7 (33.33; 14.59–56.97)		1.81 (3.14)	1.40	16.46
Mature	24 (53.33)	7 (29.17; 12.62–51.09)		1.81 (3.80)	1.49	11.96
Year			0.07			
2011	14 (31.11)	6 (42.86; 17.66–71.14)		7.32 (5.88)	1.49	16.46
2013	8 (17.78)	0 (0.00)		–	–	–
2014	8 (17.78)	3 (37.50; 8.52–75.51)		3.36 (1.53)	1.64	4.59
2017	2 (4.44)	2 (100.00; 15.81–100.00)		–	1.40	1.65
2018	2 (4.44)	1 (50.00; 1.26–98.74)		–	2.88	2.88
2023	11 (24.44)	2 (18.18; 2.28–51.78)		–	2.19	10.33

1 From Peto Peto test (Helsel, 2006).

2 Calculated for all values using robust ROS (Helsel, 2006).

3 Determined only for concentrations >LOD.

**TABLE 3:** Frequency of detection of all metabolites in urine samples collected from bottlenose dolphins (*Tursiops truncatus*) in Sarasota Bay, Florida (n=71; 2010–2019; 2022–2024).

	MBP	MBzP	MEHHP	MEHP	MEOHP	MEP	MiBP
# Above LOD	4	3	2	41	5	13	0
% Above LOD (95% CI)	5.63 (1.56–13.80)	4.23 (0.88–11.86)	2.82 (0.34–9.81)	57.75 (45.44–69.39)	7.04 (2.33–15.67)	18.31 (10.13–29.27)	0.00

**TABLE 4:** Frequency of detection, ROS mean and standard deviation, minimum, and maximum values of mono-2-ethylhexyl phthalate (MEHP) above the limit of detection (LOD; “detects”) by demography in bottlenose dolphins sampled in Sarasota Bay during 2010–2019 and 2022–2024.

Variable	Total (n=71) n (% of Total)	MEHP detects (n=41) n (%; 95% CI)	p^1^	Mean MEHP (s.d.)^2^ (µg/L)	Minimum MEHP (µg/L)^3^	Maximum MEHP (µg/L)^3^
Sex			0.30			
Female	38 (53.52)	24 (63.16; 45.99 – 78.19)		9.77 (16.20)	0.55	76.60
Male	33 (46.48)	17 (51.52; 33.54–69.20)		3.74 (9.90)	0.26	49.20
Age Class			0.50			
Immature	27 (38.03)	17 (62.96; 42.37–80.60)		3.11 (6.20)	0.26	28.40
Mature	44 (61.97)	24 (54.55; 38.85–69.61)		9.36 (16.60)	0.39	76.60
Year			0.002			
2010*	4 (5.63)	1 (25.00; 0.63–80.59)		–	0.26	0.26
2011*	2 (2.82)	0 (0.00)		–	–	–
2012*	3 (4.23)	1 (33.33; 0.84–90.57)		–	1.90	1.90
2013*	1 (1.41)	0 (0.00)		–	–	–
2014*	5 (7.04)	5 (100.00; 47.82–100.00)		40.00 (23.70)	16.00	76.60
2015*	5 (7.04)	5 (100.00; 47.82–100.00)		32.10 (4.47)	28.10	37.00
2016*	6 (8.45)	1 (16.67; 0.42–64.12)		–	1.50	1.50
2017*	9 (12.68)	5 (55.60; 21.20–86.30)		2.76 (1.91)	1.00	5.90
2018*	6 (8.45)	0 (0.0)		–	–	–
2019*	7 (9.86)	6 (85.71; 42.13–99.64)		2.62 (2.24)	0.39	6.35
2022	3 (4.23)	2 (66.67; 9.43–99.16)		–	0.64	2.76
2023	5 (7.04)	4 (80.00; 28.36–99.49)		8.87 (7.41)	3.34	19.30
2024	15 (21.13)	11 (73.33; 44.90–92.21)		5.21 (7.53)	0.71	21.30

1 From Peto Peto test (Helsel, 2006).

2 Calculated for all values using robust ROS (Helsel, 2006).

3 Determined only for concentrations >LOD.

* Urinary phthalate metabolite data obtained during 2010–2019 has previously been reported in Dziobak et al., 2021.

**TABLE 5:** Comparison of detectable urinary mono(2-ethylhexyl) phthalate (MEHP) concentrations between dolphins sampled in Barataria Bay (2011, 2013–2014, 2017–2018, 2023) and Sarasota Bay (2010–2019, 2022–2024).

	BAR MEHP	SSB MEHP	p
No. At or Above LOD	14	41	–
% Detect (95% CI)	31.11 (18.17–46.65)	57.75 (45.44–69.39)	0.005^2^
Mean (s.d.)^1^ (µg/L)	5.18 (4.66)	11.95 (16.67)	0.97^3^
Minimum^1^	1.40	0.26	–
Maximum^1^	16.46	76.60	–

1 Calculated among individuals with concentrations >LOD.

2 Calculated using Peto Peto test (χ^2^ = 7.80, df=1).

3 Calculated using Mann-Whitney U test.

## Data Availability

The datasets generated for this study can be found in the Dryad repository: https://doi.org/10.5061/dryad.pc866t20f.
